# Bibliography

**Published:** 1893-01

**Authors:** 


### Bibliography.

Die Mikroorganismen der Mundhöhle. Von W. D. Miller, Dr. Méd. et Phil., mit 134 Abbildungen im Texte und 18 Photogrammen. Leipzig: Verlag von Georg Thieme, 1892.

This very much enlarged second German edition of Professor Miller's book is but another evidence of the universally recognized value of his investigations. That the first edition should have been exhausted, and require renewal in the comparatively short period of three years, seems to be exceptional for a purely scientific publication. The explanation will, doubtless, be found in the fact that the world-wide recognition of Professor Miller's original and exhaustive investigations has made the demand for anything he may produce coextensive with his reputation.

This book has reached somewhat formidable proportions, and the question may be asked, if the author be spared to continue his labor, where the limit will be found. It is certainly true that nothing he has published in the past can be omitted, and his very great faculty of condensing would seem to have made it impossible to go much farther in this direction.

In comparison with the first German edition, there is an increase in this of one hundred and forty-nine pages, and over the American eighty-four pages. The number of new sub-chapters are consequently extensive. Most of these have been made familiar to English readers through articles in the *Dental Cosmos.* Some are to be found in the American edition.

A careful examination of this work indicates the same scientific thoroughness as those preceding.

The chapter on "Asepsis and Antisepsis in Dentistry" is one of the recent additions, and in part published in the periodical named. It has not only a practical value, but should be carefully considered, not alone for the scientific facts contained, but as a warning to those who attempt to treat pathological conditions without proper training and knowledge of the possibilities underlying all careless handling of patients.

We have not the space to follow the author through the various means adopted to sterilize glasses, instruments, etc., and the relative merits of the various agents used for the purpose.

One of the greatest difficulties met with in daily practice is the sterilization of the mouth-mirror. This has necessarily to be used constantly, and it is very important that some plan should be devised that will affect the object without injury to the glass. The suggestion for this is as follows:

"For the satisfactory and certain sterilization of the mouth-mirror, it is necessary, after a preparatory cleansing with warm water, soap, and brush, to place it in strong carbolic acid from five to ten minutes. On removing it from the carbolic acid, wash in clean water or alcohol, and dry with a clean napkin."

In addition to the article on "Micro-Organisms of Dental Decay," in the American edition, there are some further investigations made in Professor Miller's laboratory by C. Jung. The latter examined seventy-two teeth, and succeeded in isolating ten distinct species, to which he gave the name caries bacteria, A to K. Four of these—A, C, E, and K—he found nearly always constant. He developed them in various culture media with the same results as had been obtained by Miller in previous investigations.

The new chapters, as well as the additional matter distributed throughout the volume, make this book of great value, and must increase, if that be possible, the already wide and deserved reputation of the author. It is to be hoped it will be followed by an American edition, so that it may be brought within the reach of all English readers.

A Series of Questions and Answers for Dental Students, consisting of Three Parts. Freshman, Junior, and Senior Course. By Ferdinand J. S. Gorgas, M.D., D.D.S. Snowden & Cowman, Publishers. Baltimore, 1892.

These "Questions and Answers" are divided, as stated, into three parts, of sixty, ninety-two, and eighty pages, for the use of students in each of the three years now required in dental colleges of this country.

They are intended to cover all the branches supposed to be taught in a well-regulated school. That they do this better than previous attempts in this direction must be conceded; but exactly why a professor in one of our foremost colleges should have deemed it necessary to elaborate these for the use of students is not so readily explained.

The process of cramming with bare facts has been carried to an extent both in dentistry and medicine beyond what would appear justifiable to those who value thoroughness and solidity of acquirement. It is recognized that condensation in this form has a special value in some lines of work, but does not seem to be of general application. "Quiz" teaching has become an important part of the work in every well-ordered school, but this, to be of value, must not be confined to mere question and answer. The question should be the open door to the treasures beyond, and the intelligent teacher will make it the text for copious illustration of facts and principles, and in this way it becomes to the student an education. This is not possible in book-form, hence the belief that such works are not conducive to higher training.

Credit must be given to the author for covering a large field with marked ability. He has endeavored to give what he supposes may be necessary for the classes in each year, and, while the arrangement will not meet the needs of all colleges, it is, nevertheless, more nearly correct than could have been supposed possible. The long experience of Professor Gorgas as a teacher has served him well in this direction.

The failure to make a subject clear is nowhere more apparent than in the treatment of inflammation, the very foundation of pathological knowledge. He devotes nine questions to this in the first part, and it is quite certain the student will know nothing of the subject when he has answered them, nor are there any additions in the two remaining numbers.

The books as arranged will find a greater value with State Boards of Examiners than they can possibly have as aids in college work.

Lippincott's for January, 1893, opens the New Year with an exceptionally interesting number. The novel entitled "A Pacific Encounter," by Mary E. Stickney, carries the reader over to scenes on the Western coast.

A very entertaining article by Elisabeth Ballester Bates on "An Old-Time Philadelphian, Captain Charles Biddle," has a sufficiency of local flavor to find interest among the "good families" of that city.

A translation from the French of Emile Bergerat on "A Dictionary Session at the Academy" (The Immortals) is a fine piece of satire, peculiarly French in the treatment, but racy in the extreme.

"A Spanish Painter," by Colin Campbell Cooper, is a review of the work of Velasquez. In "War-Time," "Across Dug Gap," "Foils and Fencing," "An Actress and her Art," and the usual gossip of the month, complete the number.

In "Books of the Month" is a good word for our old friend and genial professor, Dr. J. E. Garretson, who, under the name of "John Darby" has entertained an ever widening circle with the philosophies of Plato, Epicurus, Spinoza, translating these into "Nineteenth Century Sense." The Messrs. Lippincott are about to bring these books out in a uniform set of six volumes.

All Around the Year 1893, a new calendar, published by Lea & Shepard, Boston, Mass., is certainly the most beautiful and artistic of these productions it has been our pleasure to see. It opens month by month with illustrations of a very young gentleman who "would a-wooing go," but "herein there lieth a tale," which is best told by the examination which it would be well for any one wishing to make a really beautiful gift to purchase. The low price of fifty cents sent to the publisher will secure one.

